# ST36 Acupuncture Alleviates the Inflammation of Adjuvant-Induced Arthritic Rats by Targeting Monocyte/Macrophage Modulation

**DOI:** 10.1155/2021/9430501

**Published:** 2021-02-27

**Authors:** Fuming Yang, Yinan Gong, Nannan Yu, Lin Yao, Xue Zhao, Shouhai Hong, Shenjun Wang, Bo Chen, Yuan Xu, Guangchang Pang, Hui Wang, Yongming Guo, Yanan Li, Yi Guo, Zhifang Xu

**Affiliations:** ^1^Acupuncture Research Center, Tianjin University of Traditional Chinese Medicine, Tianjin 301617, China; ^2^Department of Traditional Chinese Medicine, Xijing Hospital, The Fourth Military Medical University, Xi'an, Shanxi 710032, China; ^3^Acu-Moxibustion and Tuina Department, Tianjin University of Traditional Chinese Medicine, Tianjin 301617, China; ^4^Acupuncture Department, The First Affiliated Hospital of Zhejiang Chinese Medical University, Hangzhou, Zhejiang 310006, China; ^5^Tianjin Key Laboratory of Food Biotechnology, College of Biotechnology and Food Science, Tianjin University of Commerce, Tianjin 300134, China; ^6^College of Traditional Chinese Medicine, Tianjin University of Traditional Chinese Medicine, Tianjin 301617, China

## Abstract

**Background:**

Rheumatoid arthritis (RA) is a chronic systemic chronic autoimmune disease characterized by the aggregation of immune cells and secretion of cytokines in the joint synovium, causing hyperblastosis and even bone destruction. Acupuncture has been proven effective in RA treatment. This study aimed to investigate the anti-inflammatory action of acupuncture, specifically, in relation to immune cell interactions and key mediators.

**Methods:**

Rats with adjuvant-induced arthritics (AIA) were treated with manual acupuncture (MA) at *Zusanli* (ST36). Joint edema and paw withdrawal latency were monitored to observe the effects on inflammation. The levels of 24 cytokines, chemokines, and growth factors in ankle joints during the treatment (on days 1, 7, 15, and 21) were detected by multiplex immunoassay. A bioinformatics analysis based on a directed weighted mathematical model was used to construct cell communication network diagrams and identify the key cells through calculation. The monocyte/macrophage polarization in inflamed joints was investigated by detecting M1- and M2-phenotypic populations and their related cytokines.

**Results:**

ST36 MA alleviated paw edema and upregulated the nociceptive threshold of AIA rats. Several innate and adaptive immune cytokines were dynamically regulated by MA, and MA-treated rats showed a significant improvement in symptoms compared with AIA rats by day 21. The immune cell-cell communication networks were intensified with the development of RA but were significantly reduced after treatment with MA. MA was found to specifically regulate monocytes/macrophages in inflamed ankle joints ST36 MA also inhibited M1-phenotype macrophages accompanied by decreased levels of IL-1*β*.

**Conclusions:**

ST36 MA showed anti-inflammatory and analgesic effects as well as inhibition of immune cell communication networks in inflamed joints of AIA rats. Inhibiting the polarization of macrophages to the M1-phenotype in inflamed joints may be one of the key mechanisms of MA anti-inflammatory action. This research highlighted a systematic research paradigm for investigating mechanisms of acupuncture action.

## 1. Background

Inflammation is involved in the pathogenesis and progression of several diseases. Specifically, a reduction in the activity of the immune system leads to immunodeficiency, including cancer. On the other hand, overactivation of immune and inflammatory responses often contributes to the development of a variety of disorders and diseases, including rheumatoid diseases, cerebral ischemia, atherosclerosis, diabetes, and Crohn's disease [1]. Cytokines, chemokines, and related growth factors are key modulators of both homeostasis and inflammatory processes [2, 3]. The binding of these mediators to their cognate receptors triggers a cascade of cellular signaling events to regulate various cellular functions, such as proliferation, cell adhesion, apoptosis, phagocytosis, cytokine secretion, and angiogenesis [4]. The robust functioning of the immune system depends on a complex multilevel interaction network, connecting intracellular biochemical pathways, intercellular communication networks, and organ-cellular trafficking through time and space. Therefore, the investigation of the networks of inflammatory cells and secreted cytokines controlling inflammation and inflammatory disorders has attracted much attention [5, 6].

Acupuncture is a traditional medical therapy that has been used in China for more than 2500 years. As an alternative and complementary strategy, acupuncture has been recommended by the World Health Organization (WHO) to treat 16 kinds of inflammatory diseases, including rheumatoid arthritis (RA), allergic rhinitis, acute and chronic gastritis, and scapulohumeral periarthritis, and inflammatory pain [7]. The clinical symptoms of inflammatory responses, such as burning pain, redness, swelling, changes in temperature, and loss of function, are known to be alleviated by acupuncture. Acupuncture is a minimally invasive stimulation, and its anti-inflammatory effect is reported to occur by regulation of endogenous homeostatic pathways such as the hypothalamus-pituitary-adrenal axis and sympathetic and parasympathetic pathways. However, studies of the anti-inflammatory effects of acupuncture have focused primarily on specific cells and their related cytokines, rather than analyzing its potential regulatory effects on the immune network and the key cells involved [8–10].

Immune dysfunction and chronic inflammation are considered the contributing factors in the development and progression of RA. The inflammation causes the infiltration of inflammatory cells such as CD4^+^ T cell, B cells, and monocytes/macrophages. Cytokines released from these cells, such as IL-1*β*, IL-6, and TNF-*α*, activate mesenchymal cells (including synovial fibroblasts, osteoclasts, and chondrocytes) that release tissue-destroying matrix metalloproteinases, as well as stimulating the development of osteoclasts (which develop from the monocyte/macrophage lineage), which are responsible for bone degradation [11].

*Zusanli* (ST36) has been reported to be the most common acupoint used in the treatment of RA, and this acupoint is located on the Stomach Channel of Foot-Yangming and is also known as the Fu-subfu-he acupoint, which has been found to have specific anti-inflammatory effects [12]. In a previous study, we found that manual acupuncture (MA) at ST36 could downregulate several innate and adaptive immune cytokines in serum of adjuvant-induced arthritic (AIA) rats, a commonly used RA model [13]. In the present study, using the same experimental model, we have attempted to elucidate the modulatory effects of MA treatment on the immune network and its critical cells in the inflamed ankle joints of the rats for further clarifying the anti-inflammatory mechanism of MA, which will provide a new research model for investigating the therapeutic actions and mechanisms of acupuncture on inflammatory diseases.

## 2. Methods

### 2.1. Animals

Male Wistar rats (*n* = 132, the Academy of Military Medical Sciences, Tianjin) weighing 180–200 g (6 to 8 weeks old) were supplied by the Laboratory Animal Centre of Tianjin University of Traditional Chinese Medicine. All rats were allowed to acclimatize for one week prior to the experiment and were housed in a controlled environment (12 h light/dark cycle, 22 ± 2°C, 55 ± 5% relative humidity) with free access to food and water. All rats used in the present study were healthy and have never been used in other research procedures.

All experimental procedures were carried out in strict accordance with the Guidance Suggestions for the Care and Use of Laboratory Animals (formulated by the Ministry of Science and Technology of China). The protocol was approved by the Animal Care and Use Committee of Tianjin University of Traditional Chinese Medicine (permit number: TCM-LAEC2012009), and all animal handling procedures were performed in accordance with the Tianjin University of Traditional Chinese Medicine guidelines for the care and use of laboratory animals.

### 2.2. Experimental Design

**Figure 1:**
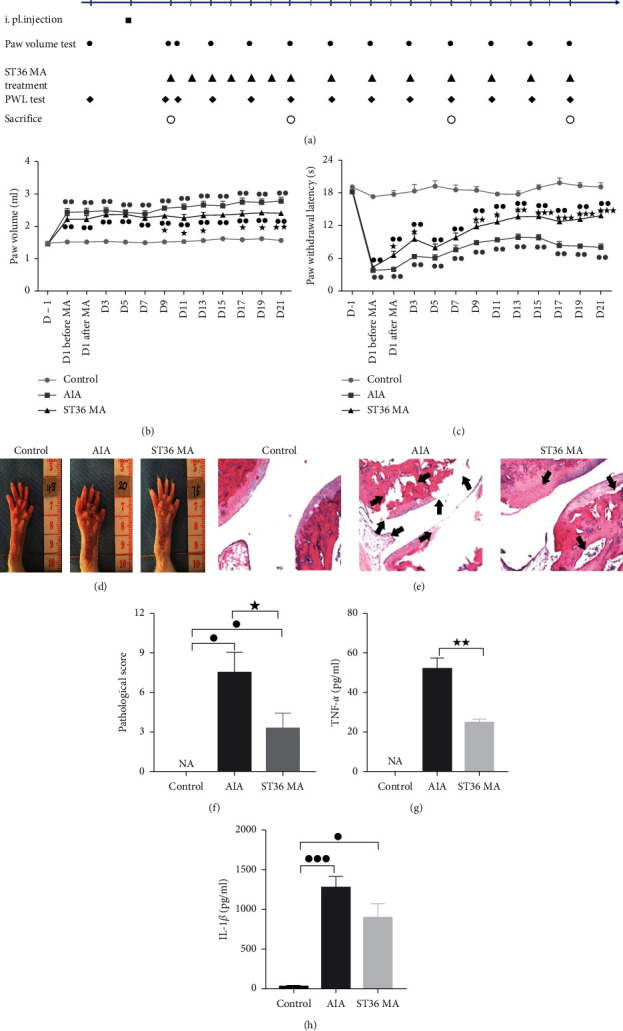
The anti-inflammatory and antinociceptive effect of ST36 MA on inflamed paws and joints of AIA rats. (a) Flowchart of acupuncture treatment and effect index measurement. Disposition represented by different symbols: ■ means CFA or saline plantar injection, ● means paw swelling volume test, ▲ means ST36 MA administration, ◆ is the PWL test, and the circles mean sacrifice and tissue collection. (b-c) The effect of ST36 MA treatment on the CFA-induced foot and ankle joint swelling in (b) and thermal hyperalgesia in (c) (*n* = 6 rats in each group). (d) Typical appearance of ankle joints in three groups on day 21. (e) Histological analyses were performed on right ankle joints (x100) stained by H&E, and different degree erosion was observed in the articular cartilage and subchondral arthritis in AIA and ST36 MA groups (black arrow). (f-g) The protein content of TNF-*α* and IL-1*β* in right ankle homogenate by ELISA. The data obtained are expressed as the mean ± SEM in control (●), AIA(■), and ST36 MA (▲)groups. ^●^*P* < 0.05, ^●●^*P* < 0.01, and ^●●●^*P* < 0.001 indicated statistical differences versus the control group; ^★^*P* < 0.05 and ^★★^*P* < 0.01 indicate statistical differences versus the AIA group.

The rats were randomly divided into three groups: the control group, AIA group, and MA group. Rats (*n* = 6 per group) received intraplantar (i.pl.) injections of complete Freund's adjuvant (CFA) or saline on day 0. The ipsilateral swelling of the ankle and paw was assessed on the day before modeling (day 1) and every other day from day 1 (3 h before model establishment or MA treatment). The paw withdrawal latency (PWL) was tested on day 1 (24 h before model establishment), days 0–7 (24 h after model establishment and 0.5 h after MA treatment), and days 8–21 (once every other day). MA treatments were given on days 1–7 (once per day) and days 8–21 (once every other day). The ipsilateral ankle joints of the rats were collected on day 21 for detecting the protein contents of TNF-*α* and IL-1*β* (*n* = 6 per group) and for evaluating the pathological damage (*n* = 4 per group). The ipsilateral ankle joints were collected on days 1, 7, 15, and 21 for detecting 24 cytokines (*n* = 6-7 per group) to analyze the cell-cell communication (CCC) network. The right paws (*n* = 4–7 per group) were collected on day 15 for assessing the macrophage subpopulations and the protein content of IL-1*β* (*n* = 4–7 per group, Figure 1(a)). All experimental tests were performed in a double-blind manner.

### 2.3. Complete Freund's Adjuvant-Induced RA Models

Rats received i.pl. injections of 0.1 ml CFA (heat-killed and dried *Mycobacterium tuberculosis* 0.85 ml paraffin oil and 0.15 ml of mannide monooleate in 1 mg/ml mixture, Sigma, San Diego, CA, USA) into the right hind paw on day 0 to induce the AIA rat model as previously described [13]. Rats in the control group received i.pl. injections of 0.1 ml PBS.

### 2.4. ST36 Manual Acupuncture Treatment

The procedure was carried out as previously described [13]. Each rat was immobilized using a soft cloth. ST36 is located 3-4 mm below and 1-2 mm lateral to the midline of the knee, and the disposable acupuncture needles (diameter = 0.35 mm, length = 25 mm, Hanyi TCM, Beijing, China) were vertically inserted into bilateral ST36 to a 3 mm depth of rats in the MA group. After *deqi*, the needles were rotated at a rate of 3 spins bidirectionally. One spin consisted of a clockwise rotation of 180 degrees and a counterclockwise rotation of 180 degrees for 2 min with a 5 min interval for a total of 28 min during an acupuncture session.

### 2.5. Measurement of Nociceptive Thresholds

The PWL was measured in the three groups using the BME-410 C heat pain stimulation instrument (Chinese Academy of Medical Sciences Institute of Biomedical Engineering, Tianjin, China) [13, 14]. Before modeling, the PWL measurements of rat right paws were determined within 16–20 s. After the rats were adapted to the new situation, the required time (s) until the withdrawal of rats' right hind paw was taken as the thermal nociceptive threshold; this was measured three times at 5 min intervals, and the mean value was recorded.

### 2.6. Measurement of Paw Swelling

The ipsilateral swelling of the ankle and paw was assessed using a self-made water displacement instrument as previously described [13].

### 2.7. Enzyme-Linked Immunosorbent Assay (ELISA)

On day 21, the rats in the three groups were weighed and anesthetized with sodium pentobarbital (50 mg/kg, i.p.), before decapitation and collection of the joints. The levels of TNF-*α* and IL-1*β* in the right ankle joints were measured using rat-specific ELISA kits (Abcam, Cambridge, UK) according to the manufacturer's instructions [13].

### 2.8. Histological Examination

The pathological examination of the ipsilateral ankle joints was conducted after the behavioral tests on day 21 [13]. Hematoxylin and eosin (H&E)-stained paraffin sections (5 *μ*m thick) were prepared, and the arthritic severity in the inflamed joints was graded, yielding the pathological score. These scores were determined in a blinded method by a single pathologist who was unaware of the animal randomization. The severity of arthritis in joints was graded from 0 to 4 according to the intensity of the lining layer hyperplasia, immune cell infiltration, and foreign body granuloma formation. The pathological scores were described as 0 = normal ankle joint, 1 = normal synovium with occasional immune cells, 2 = definite arthritis with a few layers of flat to rounded synovial lining cells and scattered mononuclear cells and foreign body granuloma formation or dense infiltration with immune cells and foreign body granuloma formation, 3 = clear hyperplasia of the synovium with three or more layers of loosely arranged lining cells and dense infiltration of immune cells and foreign body granuloma formation, and 4 = severe synovitis and foreign body granuloma formation, and even erosion of the articular cartilage and subchondral bone [13].

### 2.9. Multiplex Immunoassay

The right ankle joint was collected on days 1, 7, 15, and 21 after MA treatment for multiplex immunoassay detection of 24 inflammatory factors [13]. The total protein was extracted and stored at −80°C until used in the multiplex immunoassay. Samples (12.5 *μ*l homogenate) were applied to the magnetic bead suspension array with the Luminex® 200™ system (Invitrogen, Carlsbad, CA, USA) to measure the levels of IL-1*α*, IL-1*β*, IL-6, IL-7, IL-18, TNF-*α*, IL-2, IL-12, IFN-*γ*, IL-4, IL-5, IL-10, IL-13, IL-17, CXCL1, MCP-1, RANTES, MIP-1*α*, MIP-3*α*, GM-CSF, G-CSF, M-CSF, EPO, and VEGF. And results were calculated using Bio-Plex Manager 6.0 software (Bio-Rad Laboratories, Hercules, CA, USA).

### 2.10. Construction of the Cell-Cell Communication Network Diagram

The communication network was constructed as previously described with certain modifications [13]. Briefly, we mapped out a communication network based on the changes in cytokines content, using the documented relationships among the cytokines, immune cells, and other cells in the body. The values of the altered cytokine levels and their related cells (including secretory cells and receptors) were obtained, and the communication effect among all the cells was calculated according to the following formula:

(1) $$\begin{array}{c} \text{Ecc}=\sum_{i=1}^{n=24}SiFip, \end{array}$$

where Ecc referred to total stimulating intensity of cytokines on the objective cells; *n* referred to the sum of determined cytokines. And *S* refers to cells responsible for cytokine secretion. When one cell secretes a specific cytokine, the value of *S* would be assigned as 1; otherwise, *S* would be assigned as 0. *F* refers to the effect of one cytokine on one cell, and the value of *F* would be assigned as 1 if the cytokine had an effect on the cell; otherwise, *F* would be assigned as 0. $p=\left({\bar{x}}_{a}-{\bar{x}}_{b}\right)/{\bar{x}}_{a}$, ${\bar{x}}_{a}$, ${\bar{x}}_{b}$ is the average value of the cytokines in the different groups. Ecc values were calculated using Microsoft Excel 2016. In addition, the CCC network diagram was portrayed using Microsoft Visio 2007 software based on the Ecc values. Variations of CCC are visualized as interacting lines between cells. Additionally, the thickness of lines represents the total stimulation intensity of the cytokines on their receptor cells were quantified based on the Ecc values. E*nn* represented the communication effect of one cell on the objective cell. Immune network_sd_ = (*E*_11_ + *E*_12_ + *E*_13_ + · · + *E*_1*n*_) + · ·(*E*_*n*1_ + *E*_*n*2_ + *E*_*n*3_ + *E*_*n*4_ + · ·E_*nn*_). Then, we set up another cell output and input signal parameter calculation matrix to count the relevant parameters of the Cell_sd_ (as shown in Table 1), in which Ecc=∑_*i*=1_^*n*=24^*S*_*i*_*F*_*i*_*p*′, *p*′ represented the absolute value of change factor, $p^{\prime }=\left|{\bar{x}}_{a}-{\bar{x}}_{a}\right|/{\bar{x}}_{a}$. Output signal density_*n*_ (OSD_*n*_) represented the secretory density (OSD_*n*_=*E*_*n*1_′+*E*_*n*2_′+*E*_*n*3_′+*E*_*n*4_′+⋯+*E*_*nn*_′). For instance, OSD_1_=*E*_11_′+*E*_12_′+*E*_13_′+*E*_14_′+⋯+*E*_1*n*_′. Input signal density_*n*_ (ISD_*n*)_ in Table 1 represented the objective density (ISD_*n*_=*E*_1*i*_′+*E*_2*n*_′+*E*_3*n*_′+*E*_4*n*_′+⋯+*E*_*nn*_′), for instance, ISD_1_=*E*_11_′+*E*_12_′+*E*_13_′+*E*_14_′+⋯+*E*_1*n*_′. Cell_sd_ represented the total of absolute density including secretory and objective densities (Cell_sd_ = Abs (OSD_*n*_) + Abs (ISD_*n*_)), and Cell_sd_ with the highest value was considered as the key cell in the immune network.

### 2.11. Flow Cytometry (FCM)

Rats were anesthetized with sodium pentobarbital (50 mg/kg, i.p.), and their right paws were collected. Paw tissue fragments were cut into 1 to 2 mm pieces and digested for 1 h at 37°C with a 10 ml RPMI 1640 medium (Thermo Fisher, Shanghai, China) containing 30 mg collagenase (Sigma, Deisenhofen, Germany), 10 mg hyaluronidase (Sigma), and 0.5 ml 1 M HEPES (Sigma). Red blood cells in the digested fragments were lysed with lysis buffer (BD Bioscience, Franklin Lakes, NJ, USA). The cell suspensions were stained with CD45-PE-Cy7 (BioLegend, San Diego, CA, USA), CD11b-APC (BioLegend), CD206-FITC (Abcam), and CD86-PE (BD Bioscience) for 20 min at room temperature, then washed with PBS and diluted to 3 × 10^6^ cells/mL in 2% fetal bovine serum, and applied to a flow cytometer (ThermoFisher Attune NxT, Waltham, MA, USA) at 1500 cells/sec. The data were analyzed with Attune NxT software.

### 2.12. Statistical Analysis

All data were presented as mean ± SEM. The normality of data was confirmed, and multiple measurements at different timepoints were analyzed for paw swelling and PWL by repeated measures analysis of variance (ANOVA), and the statistical analysis of groups was analyzed by multivariate ANOVA. For dynamic cytokine levels of the joint tissue, one-way ANOVA was applied for independent samples comparing the differences between groups at each time, respectively, with SPSS software (version 19.0, IBM Corp., Armonk, NY, USA). *P* < 0.05 was regarded as statistically significant.

## 3. Results

### 3.1. The Anti-Inflammatory and Analgesic Effect of MA at ST36 in AIA Rats

The inflammatory and analgesic effect of MA on AIA rats was first evaluated. As shown in Figure 1(b)), rats in the AIA group showed persistent swelling on the ipsilateral paws and ankle joints from day 1 which lasted through 21 days after CFA injection. With MA treatment, the paw edema was reduced by day 9 and the curative effect was maintained to day 13; the benefits reappeared on days 17 and 19 and continued until day 21. The nociceptive thresholds (represented as the PWL) displayed marked decreases in the CFA-induced AIA rats on day 1 (Figure 1(c)) and lasted for 21 days. ST36 MA treatment alleviated thermal hyperalgesia from day 1 after MA treatment compared to that of the AIA rats except for days 5 and 7. Significant therapeutic effects appeared on day 15 and lasted to day 21.  Figure 1(d) shows the typical appearance of ankle joints in the three groups on day 21. Compared with the control group, the ankle joints of the AIA group showed obvious redness and swelling, while acupuncture treatment significantly alleviated these manifestations.  Figure 1(e) shows the histological structures of joints in the three groups at day 21, and Figure 1(f) shows pathological scores indicating joint damage. The joint structure was normal in the control group while the inflamed joints in AIA rats showed synovial hyperplasia, infiltration of inflammatory cells, bone destruction with high scores for inflammation, and cartilage damage. MA treatment markedly decreased the inflammation and cartilage pathological scores (Figures 1(e)–1(f)). Furthermore, there were higher levels of TNF-*α* and IL-1*β* in joint homogenates of AIA rats on day 21. ST36 MA significantly decreased the levels of TNF-*α* as well as reducing the levels of IL-1*β* compared with the AIA group (Figures 1(g)–1(h)). These results suggest that ST36 MA treatment can alleviate plantar thermal radiation pain in AIA rats, reduce foot swelling, and downregulate protein levels of proinflammatory factors (such as TNF- *α*) in inflammatory ankle joints, which verifies the anti-inflammatory and analgesic effects of acupuncture in AIA rats.

### 3.2. ST36 MA Modulated the Levels of Innate and Adaptive Cytokines in the Joints of AIA Rats

**Figure 2:**
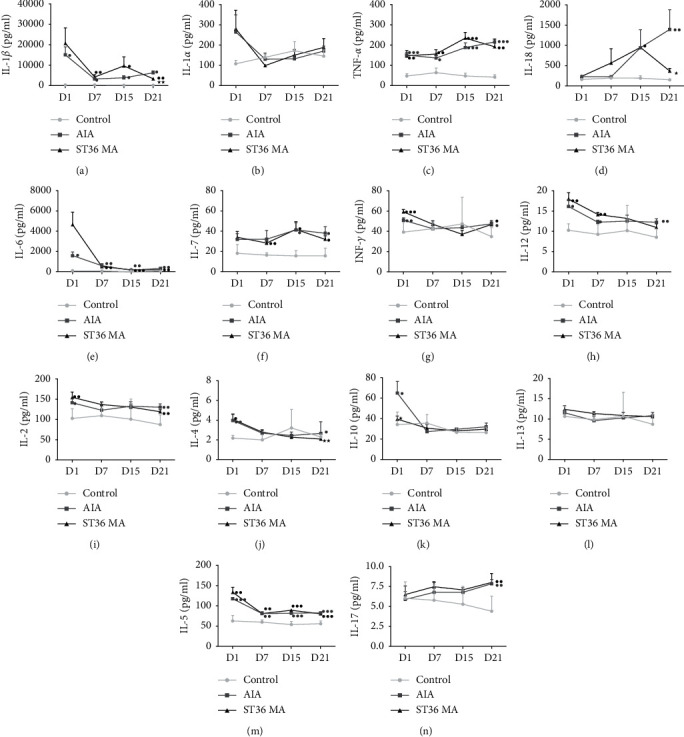
MA regulates the joint cytokines of innate immunity and adaptive immunity. Joint levels of cytokines IL-1*β* (a), IL-1*α* (b), TNF-*α* (c), IL-18 (d), IL-6 (e), IL-7 (f), IFN-*γ* (g), IL-12 (h), IL-2 (i), IL-4 (j), IL-10 (k), IL-13 (l), IL-5 (m), and IL-17 (n) on day1, day 7, day 15, and day 21after saline/CFA challenge in adult rats treated with or without MA (*n* = 6-7). The data obtained are expressed as the mean ± SEM in control (●), AIA (■), and ST36 MA (▲) groups. ^●^*P* < 0.05, ^●●^*P* < 0.01, and ^●●●^*P* < 0.001 indicated statistical differences versus the control group; ^★^*P* < 0.05 and ^★★^*P* < 0.01 indicate statistical differences versus the AIA group.

Studies have demonstrated that dysregulation of both the adaptive and innate immune response plays an important role in the pathogenesis of RA. To explain the modulation effect of MA on encountered cytokines of innate immunity, the protein levels of IL-1*β*, IL-*α*, TNF-*α*, IL-18, IL-6, and IL-7 in the right ankle joints were detected by multiplex immunoassay.  Figure 2 shows that levels of these cytokines were elevated in both the AIA and ST36 MA groups on day 1 compared to those of the control group and that modeling upregulated the levels of IL-1*β* (Figure 2(a)) and TNF-*α* (Figure 2(c)) while MA treatment upregulated the level of TNF-*α* (Figure 2(c)). On day 7, the cytokine levels in the AIA group showed a downtrend compared with those on day 1. Compared with the control group, significant differences were found in the levels of IL-1*β* (Figure 2(a)), TNF-*α* (Figure 2(c)), and IL-6 (Figure 2(e)) in the AIA group. However, secondary flares of IL-1*β* (Figure 2(a)), TNF-*α* (Figure 2(c)), IL-18, and IL-7 (Figure 2(f)) release were observed on day 15. On day 21, the levels of IL-1*β* (Figure 2(a)), TNF-*α* (Figure 2(c)), IL-18 (Figure 2(d)), and IL-6 (Figure 2(e)) were lower compared to those of the control group. However, the levels of these cytokines in the AIA group were increased at each timepoint (day 1, day 7, and day 15) compared with those of the control group. From day 1 to day 15, levels of the innate immune cytokines in the ST36 MA group were essentially the same as those in the AIA group, which also increased on day 1, decreased on day 7, and increased again on day 15. On day 21, the levels of IL-1*β*, TNF- *α*, IL-18, and IL-7 in the ST36 MA group were lower than those in the AIA group, with statistical differences found in IL-1*β* (Figure 2(a)) and IL-18 (Figure 2(d)).

Within the 21 days of MA treatment, the adaptive immune factors in the control group showed no obvious fluctuation among the four timepoints. In both the AIA and ST36 MA groups, the Th1 cell-secreted cytokines increased on the first day after modeling, with significant differences observed in INF-*γ* (Figure 2(g)), IL-12 (Figure 2(h)), and IL-2 (Figure 2(i)) in both groups. However, the levels of these cytokines in the AIA group showed a downtrend at the other three timepoints. Th2 cell-related cytokines such as IL-4, IL-10, IL-13, and IL-5 showed a similar rising trend on day 1 compared with the control group in both the AIA and ST36 MA groups, with statistical differences observed in IL-4 (Figure 2(j)) and IL-5 (Figure 2(m)). Acupuncture treatment significantly downregulated the level of IL-4 on day 21 (Figure 2(j)) compared with the AIA group. Furthermore, IL-17 (released by Th17 cells) did not increase on the first day after modeling in the AIA and ST36 MA groups but gradually increased over time, and both groups showed a significant difference on day 21 compared with the control group (Figure 2(n)). These results suggest that acupuncture has a regulatory effect on both the innate and adaptive immune systems in inflamed joints in AIA rats, accompanied by anti-inflammatory actions on day 21.

### 3.3. The Effects of ST36 MA on the Levels of Chemokines and Growth Factors in the Joints of AIA Rats

**Figure 3:**
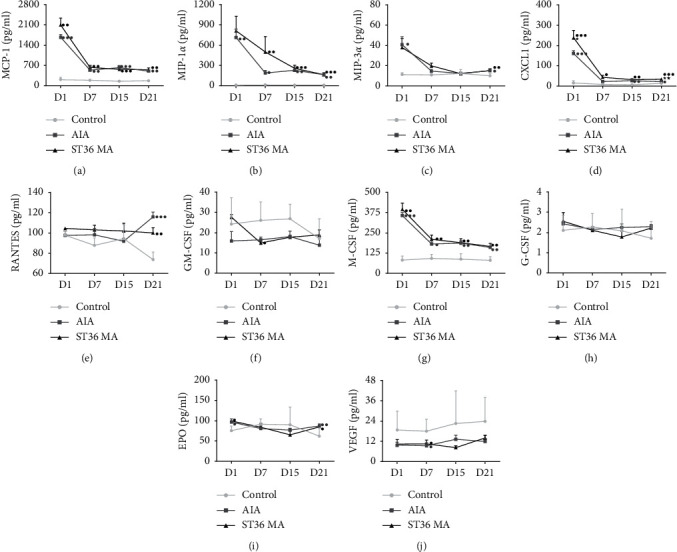
The effects of ST36 MA on the levels of chemokines and growth factors in the joints of AIA rats. Joint levels of MCP-1 (a), MIP-1*α* (b), MIP-3*α* (c), CXCL1 (d), RANTES (e), GM-CSF (f), M-CSF (g), G-CSF (h), EPO (i), and VEGF (j) on day1, day 7, day 15, and day 21after saline/CFA challenge in adult rats treated with or without MA (*n* = 6-7). The data obtained are expressed as the mean ± SEM in control (●), AIA (■), and ST36 MA (▲) groups. ^●^*P* < 0.05, ^●●^*P* < 0.01, and ^●●●^*P* < 0.001 indicated statistical differences versus the control group; ^★^*P* < 0.05 and ^★★^*P* < 0.01 indicate statistical differences versus the AIA group.

Chemokines promote the dynamic migration of immune cells, and they may influence the inflammatory response. Therefore, we investigated several chemokines, including CXCL1, MCP-1, MIP-3*α*, RANTES, and MIP-1*α*. Additionally, we ran several tests of growth factors. On day 1, levels of chemokines such as MCP-1 (Figure 3(a)), MIP-1*α* (Figure 3(b)), MIP-3*α* (Figure 3(c)), and CXCL1 (Figure 3(d)) in the AIA group were significantly higher than those in the control group. On day 7, the levels of MCP-1, MIP-1*α*, MIP-3*α*, and CXCL1 in the AIA group were lower than on day 1 but still higher than those in the control group, and statistical differences were found with MCP-1 (Figure 3(a)) and MIP-1 *α* (Figure 3(b)). On day 15, chemokine levels were relatively stable, and statistical differences were found in MCP-1 (Figure 3(b)), MIP-1*α* (Figure 3(b)), and CXCL1 (Figure 3(d)). On day 21, compared with the control group, significant differences were found in the levels of MCP-1 (Figure 3(a)), MIP-1*α* (Figure 3(b)), MIP-3*α* (Figure 3(c)), CXCL1 (Figure 3(d)), and RANTES (Figure 3(e)). During the detection period, variations in the levels of these five chemokines in the ST36 MA group were similar to those in the AIA group. Compared to the AIA group, CXCL1 in the ST36 MA group increased significantly on day 1 and 21 (Figure 3(d)). The level of M-CSF in the AIA group was upregulated significantly at the four timepoints especially on day 1 (Figure 3(g)) while the level of VEGF was downregulated on day 7 (Figure 3(j)). The level of EPO was increased on days 1 (Figure 3(i)) and 21 (Figure 3(i)). The variation trend of M-CSF in the ST36 MA group was essentially the same as that in the AIA group, being upregulated at each of the four timepoints (Figure 3(g)). At each timepoint, there were no significant differences among all the growth factors between the acupuncture group and the AIA group. The above experimental results suggested that acupuncture has no significant regulatory effect on chemokines and growth factors.

### 3.4. MA Regulated the Immune Cell-Cell Communication Network in Inflamed Joints by Targeting Macrophages

**Figure 4:**
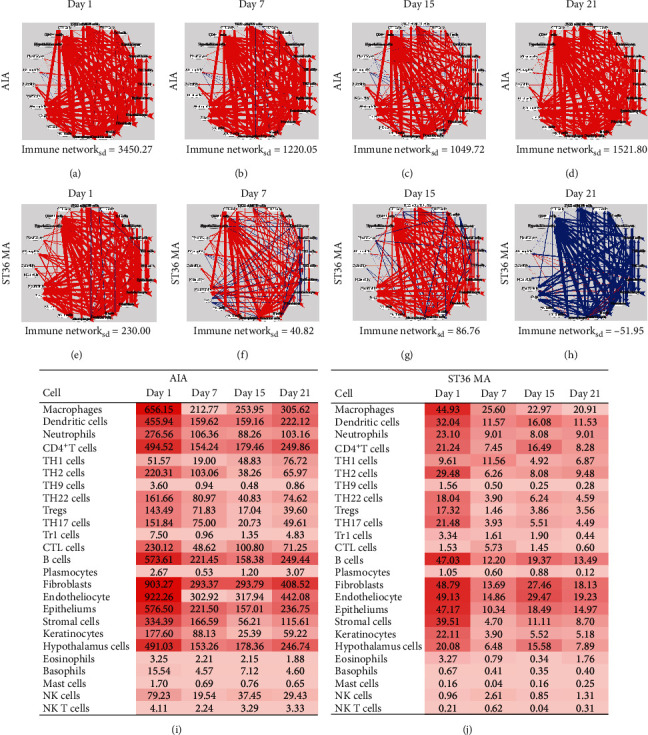
MA regulates cell-cell communication network. (a–h) The cell-cell communication networks were mapped in AIA condition (a–d), and AIA rats were treated with MA condition (e–h) on day 1, 7, 15, and 21. Immune networks represents the whole immune network density between cells in indicated condition. (i-j) Heat maps of total density of involved cells in the right ankle of AIA rats (i) and AIA rats were treated with MA (j). Cells represent the total density including secretory and objective densities, and cells with high value (deep color) were considered as the key cell in the immune network.

To identify the key target cells of MA, we drew a CCC network diagram at the four timepoints (Figures 4(a)–4(h)), in which the red lines represent the enhancement and the blue lines the reduction in cell-to-cell interactions. The thicker the lines, the stronger the effect. Lines pointing from one cell to another indicate that the signal intensities are altered by interventions from one cell to another. A positive or high network value represents strong communication intensity. Modeling was found to enhance cell-to-cell communication, an effect that gradually diminished over the first 15 days (cell network density was 3450.27 on day 1, 1220.05 on day 7, and 1049.72 on day 15). The intensity of cell communication on day 21 (cell network density of 1521.80) was higher than that on day 15. As an external stimulus, acupuncture enhanced the immune network of the inflammatory site on day 1 (cell network density was 230.00) compared with the AIA condition, resulting in an increased intensity of immune actions on target organs. In the following days 7 and 15, the level of cytokines in the acupuncture group was similar to that at day 1 (cell network density at days 7 and 15 was 40.82 and 86.76, respectively), and the intensity of the immune network was decreased on day 21 (cell network density was −51.95) when the acupuncture anti-inflammatory actions took effect. Figures 4(i)–4(j) show that modeling activates mainly endothelial cells, fibroblasts, macrophages, epithelial cells, and CD4^+^T cells, essentially in accordance with the pathological characteristics of RA. At each time-point, macrophages were one of the key cell types activated by acupuncture treatment. These findings lay the foundations for further exploration of acupuncture's anti-inflammatory effects.

### 3.5. Effect of ST36 Acupuncture on Macrophage Polarization in Inflamed Joint and Paw Tissue

**Figure 5:**
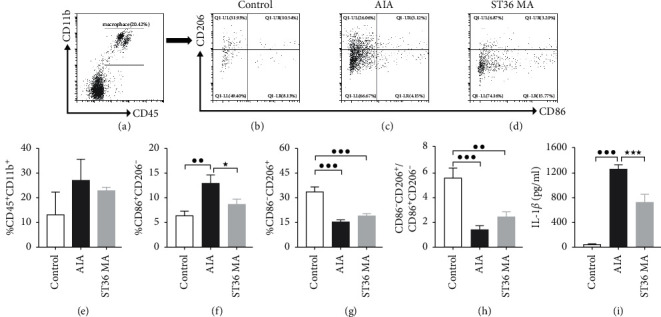
The effect of MA at ST36 on the macrophage polarization of AIA rats. (a–c) FCM charts of cell populations of M1 and M2 phenotypes by staining panel CD45^+^CD11b^+^CD86^+^CD206^+^. The presentative images of microphage subpopulations (M1/M2) in control (b), AIA (c), and ST36 MA (d) groups. (d–f) The quantitative analysis of total macrophage (CD45^+^CD11b^+^) in (d), CD45^+^CD11b^+^CD86^+^CD206^−^dominant M1 population in (e), and CD45^+^CD11b^+^CD206^+^ CD86^−^ dominant M2 population in (f). (g) The ratio of M1/M2 population. (h) Protein level of IL-1*β* in affected joint at day 15. Values are means ± SEM of each group. ^●^*P* < 0.05, ^●●^*P* < 0.01, and ^●●●^*P* < 0.001 indicated statistical differences versus the control group; ^★^*P* < 0.05 and ^★★★^*P* < 0.001 indicate statistical differences versus the AIA group.

Since acupuncture treatment showed definite amelioration of joint swelling and thermal radiation pain by day 15 and the therapeutic effect lasted to day 21, we further investigated MA modulation of monocytes/macrophage functioning for a 15-day time course. Macrophage polarization has been found to be crucially involved in the pathogenesis of RA [15–17]. M1 macrophages express proinflammatory cytokines and mediators while M2 macrophages secrete factors that suppress inflammation and promote tissue repair [18, 19]. We, therefore, investigated whether macrophage polarization was involved in the anti-inflammatory action of ST36 MA, measuring the populations of M1/M2 macrophages on day 15 following CFA injection. Figures 5(b) and 5(f) show a slightly increased population of CD45^+^CD11b^+^ macrophages in the inflamed group compared to the saline-injected controls. Interestingly, the proportion of M1 macrophages (CD45^+^CD11b^+^CD86^+^) was increased in the AIA group (Figures 5(d) and 5(g)) and was slightly reversed by ST36 MA (Figures 5(e) and 5(g)). The levels of M2 macrophages were decreased in the AIA and ST36 MA groups, but there was no significant difference between them (Figures 5(d), 5(e), and 5(h)). Meanwhile, MA treatment slightly reversed the proportion of M2/M1 macrophages (CD86^−^CD206^+^/CD86^+^CD206^−^, Figure 3(i)). The results indicate that AIA rats show increased numbers of M1 macrophages in inflammatory tissue, while ST36 MA treatment resulted in a normalization of the M1 population and a slight increase in the M2 phenotype ratio. The protein levels of the joint M1-phenotype cytokine IL-1*β* were higher in AIA rats than those in controls, while ST36 MA treatment reversed this elevation (Figure 5(j)). These results suggest that acupuncture can promote macrophage polarization to the M2 phenotype in arthritis sites.

## 4. Discussion

In 2002, RA was recommended as one of the indications for acupuncture by the WHO. AIA rats are frequently used as an inflammatory pain model for investigating the actions of acupuncture [20]. In the present study, one day after CFA injection, AIA rats showed persistent inflammatory symptoms in ankle joints, including redness, swelling, and pain. The analgesic effect of acupuncture appeared on day 1 of treatment and lasted until 21 days. However, joint detumescence appeared within one week of MA treatment and showed a significant difference from day 9. Thus, the anti-inflammatory effects of MA appeared relatively late. Moreover, the effect of MA was related to time: the rise in the pain threshold became increasingly evident over the treatment period and the detumescence of the limbs tended to be stable from day 9. The alleviation of symptoms showed some variation at the different timepoints which was considered to be due to individual differences and fluctuations in the rate of paw swelling and PWL of the AIA models. In addition, the pathological scores of the ankle joints at day 21 demonstrated that acupuncture could alleviate the infiltration of inflammatory cells, as well as cartilage and bone destruction in the joints to a certain extent, together with reducing the levels of proinflammatory factors such as IL-1*β* and TNF-*α* [13]. Thus, it is speculated that the anti-inflammatory and analgesic effects of MA are not synchronous, and the early analgesia may be due to the inhibition of the ascending pain pathway and the promotion of the pain descending inhibitory system, while repeated acupuncture may exert its anti-inflammatory effect by inhibiting the peripheral and central sensitization of pain process.

It is apparent that the network between cytokines and cells is of fundamental importance in RA pathogenesis. Moreover, RA is a slowly developing disorder with changing pathological characteristics over time that correlate with dynamic changes of cytokine levels in joints [21]. Therefore, the application of CCC network analysis is indispensable in studying the anti-inflammatory effects of MA in RA treatment as it yields a comprehensive, dynamic picture of acupuncture regulation [22]. Our previous research has confirmed this point by identifying multiple immune mediators, such as innate and adaptive immune factors, growth factors, and chemokines, in blood samples from AIA rats, confirming that RA is characterized by systemic immune network imbalance and dysfunction. It is noteworthy that MA enhanced the intensity of connection of these immune networks in the earlier stage of RA and restored the network to a normal level in the later stage, which may contribute to the anti-inflammatory action of MA [13].

In the current study, we have tried to clarify the local immune network in the inflamed joints and paws of AIA rats, and the effects of MA on this immune network imbalance by measuring the levels of 24 immune mediators and using bioinformatic CCC network analysis. On the first day after modeling, both the innate and adaptive immune factors in the AIA group increased significantly, and the communication intensity of the immune network was significantly enhanced in these rats on days 1, 7, 15, and 21 compared to the control rats. Compared with the network on the first day, the enhancement of immune network communication intensity in the modeled rats was relatively weaker on days 7, 15, and 21, which could be explained by a gradual reduction in immune intensity after the initial acute inflammation caused by CFA injection. The cells activated in the modeling state included endothelial cells, fibroblasts, monocytes/macrophages, B cells, and CD4^+^ T cells, consistent with the pathological characteristics of RA. Acupuncture at ST36 can enhance the immune network of inflammatory joints on day 1 after modeling, not only causing a local inflammatory reaction, but also systemic stress, resulting in an increase in the intensity of the immune network of target organs. In the subsequent days 7 and 15, the intensity was slightly upregulated by MA. On day 21, the reduction in the immune network intensity was accompanied by the decrease of most innate immune cytokines (IL-1*α*, IL-1*β*, TNF-*α*, IL-18, IL-6, and IL-7) and some adaptive immune cytokines, indicating that the reduction in immune network intensity by acupuncture may be mediated through the regulation of innate immunity (Figures 2–4). What is important is that by calculating the densities of secretory and receptor cells at the different timepoints, we observed that the key cells affected by MA were macrophages. Although 24 major cytokines, chemokines, and growth factors were used to establish the CCC networks, the data are nevertheless limited and, in the future, we intend to compile more detailed and comprehensive networks to analyze the anti-inflammatory actions of acupuncture using proteomics and related techniques.

In nonspecific, or innate, immunity, local microenvironmental changes and different stimuli will lead to different macrophage responses, resulting in distinct M1 and M2 functional phenotypes [23]. The degree and type of synovial tissue macrophage polarization in patients should probably be considered a diagnostic marker of RA [24]. In RA progression, macrophages function through polarizing into the proinflammatory ‘M1' phenotype, leading to the production of toxic effector molecules such as reactive oxygen species and nitric oxide, and proinflammatory cytokines, which induce cartilage and bone erosion. Macrophages polarize to the ‘M2' phenotype, characterized by the expression of anti-inflammatory cytokines such as TGF-*β* and IL-10, and metabolism of arginine to ornithine and polyamine intermediates, to promote healing [25]. Therefore, to further clarify the regulatory effects of acupuncture on macrophage polarization, we analyzed the effect of acupuncture on macrophage subsets on day 15 when the acupuncture has taken effect (using the M1 and M2 cell surface markers, CD45, CD11b, CD86, and CD206, as markers). It was found that the number of M1 macrophages decreased and the M2/M1 ratio increased in the ST36 MA group, with decreasing levels of the M1-specific cytokine IL-1*β*. Thus, we speculate that acupuncture can inhibit M1 macrophage polarization in RA. Studies have shown that acupuncture can trigger macrophage polarization in other diseases. For instance, it has been found that electroacupuncture reduced the proportion of M1 macrophages and the levels of TNF-*α*, IL-1 *β*, and IL-6 in rats with spinal cord injury and also increased the amount of IL-10 and M2 macrophages and upregulated the expression of the M2 markers CD206 and NT-3 [26, 27].

By reviewing the published literature on acupuncture treatment of RA, it was found that ST36 was the most widely used among the 196 commonly used acupoints [28]. ST36 is also frequently selected in the clinical treatment of inflammatory pain [29]. According to traditional Chinese medicine, ST36 has the effect of tonifying and replenishing qi and is often used to treat lower limb disorders because it is localized in the lower limb. In addition, acupuncture at ST36 has also been demonstrated to have definite anti-inflammatory action [12, 30, 31]. For instance, electroacupuncture at ST36 can activate the vagus nerve projecting to the adrenal medulla, thus promoting peripheral dopaminergic anti-inflammatory effects which have been used in the effective treatment of sepsis [12]. How acupuncture at ST36 regulates the polarization of macrophages is an important question. Our previous study has found that MA at ST36 can promote the release of immune-related cytokines (such as IL-1*β*, IL-6, TNF-*α*, TLR4, and HMGB1), several chemicals (such as histamine, 5-hydroxytryptamine, substance P, and *x*-endorphin) from the acupoint, which may send information to the central nervous system (CNS) by activating peripheral nerve endings or receptors. The CNS may then promote anti-inflammatory actions via neuroimmune innervation or neuroendocrine regulation. Meanwhile, the substances from the acupoint may also enter the circulation resulting in anti-inflammatory actions by humoral immune regulation [32].

Macrophage polarization is regulated by a variety of signaling molecules and their pathways. Acupuncture has been proven to regulate the following signaling pathways and transcription factors: the phosphoinositide 3-kinases/Akt signaling pathway [33, 34], the Notch signaling pathway [35], Janus kinase-signal transducers and activators of transcription signaling pathways [36], the TGF-*β* signaling pathway [37], and the TLR4/NF-*κ*B signaling pathway [38], and all of which are important for macrophage polarization. Furthermore, it has been shown that the microRNAs miR-155, miR-125a-3p, miR-132, miR-27a, miR-193b, miR-29b, miR-222, and miR-26a-2 participate in the polarization of M1/M2 macrophages [39]. Different CD4^+^ T cell subsets may also have distinct effects on the differentiation of monocytes/macrophages into different subsets induced by cell contact and cytokine dependence. During inflammation, Th1 and Th17 cells produce cytokines such as IFN-*γ*, TNF, and GM-CSF, which mediate M1 macrophage polarization. The Th2 cytokines IL-4 and IL-13 as well as regulatory T (Treg) cells drive macrophages to the M2-phenotypic polarization [40]. In addition, autonomic nerves could regulate the polarization of lymphocytes and macrophages. For instance, stimulation of the splenic vagus nerve could activate *α*-7 nicotinic acetylcholine receptors on CD4^+^CD25^+^Foxp3^+^ Treg cells and enhance the immunosuppressive effect of the Treg cells [41]. Wang et al. and other studies have confirmed that electroacupuncture can alleviate hypersensitivity by regulating the balance of Th1/Th2 cells [42]. Therefore, we speculate that acupuncture may regulate T cell polarization and subsequent macrophage polarization through autonomic nerves to control RA, mediating both anti-inflammatory and analgesic effects. This hypothesis requires further investigation.

## 5. Conclusions

In conclusion, MA at bilateral ST36 showed anti-inflammatory and analgesic effects on RA rats and could dynamically regulate the local cellular communication network of the inflamed joints. The key cell involved in the effects of acupuncture in joints is the monocyte/macrophage, which could inhibit M1 macrophage formation, indicating that acupuncture can promote the polarization of macrophages to the M2 phenotype at the site of inflammation. This study demonstrates a scientific basis for acupuncture treatment of RA and a new research model for the regulatory role of acupuncture in inflammatory disease.

## Figures and Tables

**Table 1:** Cell output and input signal parameter calculation matrix.

	Cell 1	Cell 2	Cell 3	…	Cell *n*	Output signal density
Cell 1	*E*_11_′	*E*_12_′	*E*_13_′	…	*E*_1*n*_′	**OSD**_**1**_
Cell 2	*E*_21_′	*E*_22_′	*E*_23_′	…	*E*_2*n*_′	**OSD**_**2**_
Cell 3	*E*_31_′	*E*_32_′	*E*_33_′	…	*E*_3*n*_′	**OSD**_**3**_
…	…	…	…	…	…	
Cell *n*	*E*_*n*1_′	*E*_*n*2_′	*E*_*n*3_′	…	*E*_*n*4_′	**OSD**_***n***_
**Input signal density**	**ISD**_**1**_	**ISD**_***n***_	**ISD**_***n***_	**ISD**_***n***_	**ISD**_***n***_	

## Data Availability

The data used to support the findings of this study are available from the corresponding author on request.

## Abbreviations

AIA: Adjuvant-induced arthritic
Arg-1: Arginase 1
CCC: Cell-cell communication
CFA: Complete Freund's adjuvant
ELISA: Enzyme-linked immunosorbent assay
FCM: Flow cytometry
H&E: Hematoxylin and eosin
i.pl.: Intraplantar
LPS: Lipopolysaccharide
MA: Manual acupuncture
iNOS: Nitric oxide synthase
PWL: Paw withdrawal latency
RA: Rheumatoid arthritis
WHO: World Health Organization.

## Additional Points

(1) ST36 acupuncture inhibits joint inflammation in AIA rats. (2) ST36 acupuncture modulates immune networks in the inflamed joints of AIA rats. (3) The key cells mediating the actions of ST36 acupuncture on AIA joints are monocytes/macrophages.
